# Prognostic value of serum lipids in newly diagnosed acute promyelocytic leukemia

**DOI:** 10.3389/fonc.2025.1522239

**Published:** 2025-02-18

**Authors:** Shijie Wang, Qian Wang, Shuxin Lv, Ling Qin

**Affiliations:** Department of Hematology, The First Affiliated Hospital, and College of Clinical Medicine of Henan University of Science and Technology, Luoyang, China

**Keywords:** acute promyelocytic leukemia, dyslipidemia, triglycerides, low-density lipoprotein, prognostic markers

## Abstract

**Background and purpose:**

Dyslipidemia has been linked to acute promyelocytic leukemia (APL), with abnormal lipid metabolism observed during treatment. However, its role in APL pathogenesis remains unclear. This study investigates the relationship between serum lipid levels and clinical features, risk stratification, bleeding tendency, and prognosis of newly diagnosed APL patients, focusing on the role of the PTK2 gene in regulating lipid metabolism and its potential as a therapeutic target.

**Materials and methods:**

We analyzed 90 newly diagnosed APL patients and 99 controls. Statistical analyses, including logistic regression, survival analysis, and protein-protein interaction (PPI) network, were used to assess lipid correlations with APL. Subgroup analyses explored specific clinical impacts, and functional experiments validated PTK2’s role in lipid metabolism.

**Results:**

Elevated triglycerides (TG) were positively associated with high-risk APL, while reduced high-density lipoprotein cholesterol (HDL-C) levels correlated with lower risk. Low-density lipoprotein cholesterol (LDL-C) was an independent prognostic marker, with lower levels linked to poorer outcomes. PTK2 expression significantly promoted APL cell proliferation, migration, and lipid metabolism, highlighting its role in APL pathogenesis. PTK2 regulates lipid metabolism-related factors, such as LDL and fibrinogen, through molecular pathways.

**Conclusion:**

Dyslipidemia is closely related to APL, with TG and LDL-C levels being key prognostic indicators. PTK2 plays a crucial role in lipid metabolism regulation and APL progression, providing a new molecular basis for risk assessment and targeted therapy. These findings offer potential biomarkers for early diagnosis and personalized treatment strategies.

## Graphical Abstract

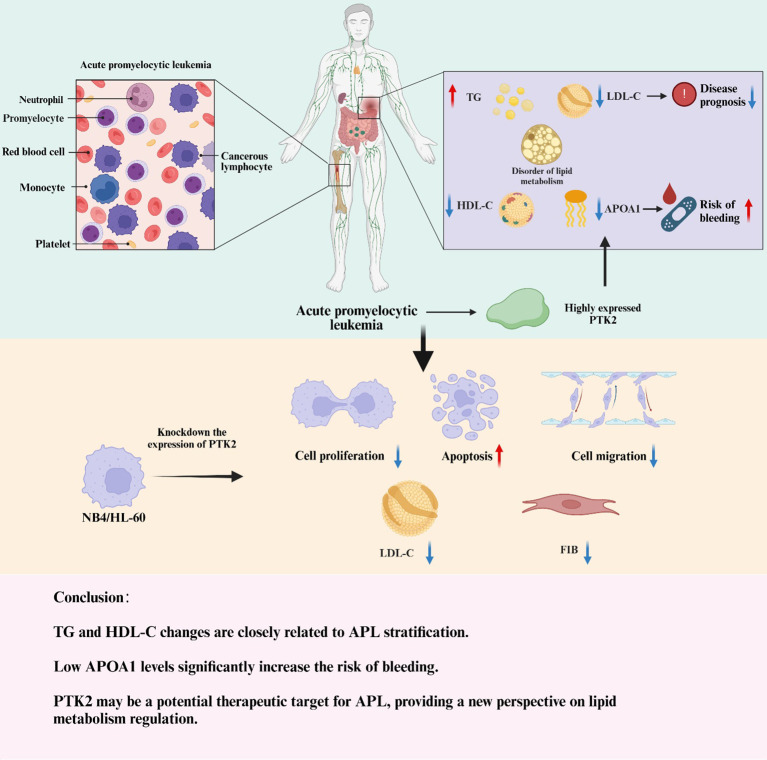

## Introduction

Acute Promyelocytic Leukemia (APL), a distinctive subtype of acute myeloid leukemia (AML), is defined by the excessive proliferation of promyelocytes within the granulocytic lineage in the bone marrow. The underlying molecular mechanism of APL involves the aberrant chromosomal translocation resulting in the formation of the PML-RARα fusion gene (1, 2). The protein resulting from this fusion gene disrupts the regular cell differentiation process, leading to the buildup of promyelocytes in the bone marrow, and facilitates the proliferation and survival of leukemia cells. The administration of all-trans retinoic acid (ATRA) and arsenic trioxide markedly enhances complete response rates and long-term survival in APL (1). Nonetheless, APL patients continue to experience elevated mortality rates and are prone to bleeding complications (3). Consequently, timely diagnosis and preventive strategies assume additional significance in managing APL.

Numerous studies have demonstrated the implication of aberrant lipid metabolism in the pathogenesis of several cancers (4–7). Such dysregulated lipid metabolism influences inflammatory responses and immune regulation, impacting cancer initiation and progression (8). Biomarkers associated with lipid metabolism were utilized to aid in the diagnosis and treatment of certain solid tumors (6, 9). A study conducted in Korea demonstrated that individuals with reduced levels of high-density lipoprotein (HDL) were at a higher risk of developing hematological malignancies (10). The association between multiple myeloma and disrupted lipid metabolism in hematological malignancies has garnered significant attention for its implications in diagnosing and treating multiple myeloma (11).

Similarly, abnormal lipid metabolism has been linked to lower survival rates in AML and is considered a pertinent target for AML treatment (12). In newly diagnosed APL, hypertriglyceridemia has been observed to be closely associated. Triglycerides and cholesterol, recognized as signaling molecules indicative of tumor growth, have been identified to play specific roles in cancer development by transmitting signals that support tumor progression and contribute to distinct lipid components (13). Nevertheless, no correlation study has been conducted on other lipid indexes. Abnormal serum lipid levels may be linked to the diagnosis and prognosis of APL. This study aims to investigate the mechanisms underlying hypertriglyceridemia in newly diagnosed APL patients, offering a new therapeutic approach for treating APL.

LDH is a glycolytic enzyme that plays a crucial role in cellular energy metabolism and is ubiquitously expressed in various body tissues. Extensive research on LDH has revealed its presence in many solid tumors, serving as an indicator of poor prognosis. Elevated levels of LDH signify a more significant tumor burden and have emerged as a prevalent biomarker in the field of oncology (14). Currently, there is limited focus on targeting LDH in hematologic tumors. This study not only incorporates the analysis of serum lipid indexes but also conducts a correlation analysis between LDH levels and standard clinical parameters in newly diagnosed cases of APL. Furthermore, the genes contributing to abnormal lipid metabolism were examined using the GEO database. Subsequently, an enrichment analysis was conducted to identify potential targets for the future diagnosis and prevention of APL. PTK2, also known as protein tyrosine kinase 2, is a non-receptor tyrosine kinase encoded by the human cell’s genes. It is widely expressed in various tissues, including the digestive system, central nervous system, eyes, urinary reproductive system, etc. PTK2, through its tyrosine kinase activity, participates in processes such as cell adhesion, migration, and proliferation, playing a positive regulatory role in cell population proliferation, ubiquitin-dependent protein degradation processes, and protein phosphorylation. PTK2 plays a role in vascular morphogenesis, neuronal generation, and tyrosine autophosphorylation processes, and various factors, including extracellular matrix, growth factors, and intracellular signaling molecules, regulate its activity. Abnormal functioning of PTK2 is associated with various diseases, including leiomyoma, small-cell lung cancer, and pulmonary arterial hypertension (15). Recent studies have found that PTK2 has been found to regulate cell proliferation and migration in various cancers, making it a potential target for drug development and disease treatment (16). In addition, abnormal activation of PTK2 affects cell adhesion and migration and plays an important role in metabolic regulation in tumor cells (17). In acute myeloid leukemia (AML), abnormal expression of PTK2 is associated with poor prognosis and treatment resistance (18); therefore, exploring the role of PTK2 in APL is of great significance for understanding the relationship between lipid metabolism disorders and APL.

In recent years, the advancement of molecular biology and cell biology technologies has provided new methods for studying the relationship between cancer and lipid metabolism. Protein-protein interaction (PPI) network analysis, gene enrichment analysis, and techniques such as qPCR, Western blot, and ELISA for gene expression have gradually been applied to explore the role of lipid metabolism dysfunction in cancer pathogenesis. These technologies reveal the functions of genes or proteins and provide a molecular-level understanding of the relationship between lipid metabolism and tumor cell behavior (19). Protein-protein interaction (PPI) network analysis typically utilizes RNA-Seq, expression profiling arrays, or proteomic analysis to identify a series of differentially expressed genes or proteins between different sample groups. Subsequently, potential interactions between encoded proteins are explored through a series of database searches, and a protein interaction network is constructed to describe the relationships among these genes or proteins, such as physical contact, targeted modulation, etc., ultimately elucidating meaningful molecular regulatory networks within organisms (20). Despite significant achievements in solid tumor research, these advances have been relatively scarce in applying to hematologic malignancies such as APL. To address this issue, this study utilizes a variety of aforementioned technologies, combined with PPI network analysis, to identify the key gene PTK2 related to lipid metabolism in APL, aiming to systematically analyze its role in lipid metabolism abnormalities in APL and provide new insights into the pathogenesis of the disease.

This study explores the relationship between lipid metabolism abnormalities in APL patients (such as changes in levels of TG, LDL-C, etc.) and their risk stratification, bleeding tendencies, and prognosis; validates the role of the PTK2 gene in the pathogenesis of APL and its regulatory function in lipid metabolism through gene expression and functional experiments; identifies lipid metabolism indicators and the PTK2 gene as potential biomarkers for risk stratification and prognosis assessment in APL, providing targets for future personalized therapy and new drug development. Specifically, we included 90 newly diagnosed APL cases and 99 healthy controls and analyzed the association between serum lipid indicators and APL clinical features using non-parametric tests, logistic regression, and survival analysis. Furthermore, through experiments such as qPCR, Western blot, and ELISA, we analyzed the expression of the PTK2 gene in APL cells and its effects on cell proliferation, migration, apoptosis, and lipid metabolism. The scientific significance of this study lies in revealing the role of disrupted lipid metabolism in APL and its regulatory relationship with PTK2, providing a new perspective for the molecular mechanism research of APL; in terms of clinical applications, the study results are expected to provide potential biomarkers and targeted treatment targets for early diagnosis, bleeding risk prediction, and formulation of personalized treatment strategies for APL patients, thereby improving the prognosis of APL patients to a certain extent.

## Materials and methods

### Patient source

This study included 90 patients diagnosed with acute promyelocytic leukemia (APL) from 2014 to 2024 at the First Affiliated Hospital of Henan University of Science and Technology. The diagnoses of all patients were confirmed based on the “Chinese APL Diagnosis and Treatment Guidelines” and the “World Health Organization (WHO) Classification of Hematologic Diseases” (21). The study included newly diagnosed APL patients and excluded patients with the following conditions: ① other malignant tumors or comorbidities (including known diseases that can cause abnormal lipid levels, such as hypertension, diabetes, cardiovascular diseases, thyroid dysfunction, etc.); ② use of medications that may affect the results or have undergone specific treatments, use of medications affecting lipid levels in the past 3 months (such as statins, fibrates, etc.); ③ special populations, such as pregnant women, minors, patients with mental illness; ④ factors affecting compliance or follow-up, such as patients with incomplete clinical data. The study ultimately included 90 APL patients and recruited 99 healthy control individuals. The entire inclusion process is shown in **Supplementary Figure S1**.

The inclusion criteria for the healthy control group were: ① no autoimmune diseases, malignant tumors, or comorbidities (including known diseases that can cause abnormal lipid levels, such as hypertension, diabetes, cardiovascular diseases, thyroid dysfunction); ② not having suffered from any gastrointestinal-related diseases in the past 3 months, no use of medications affecting lipid levels (such as statins, fibrates); ③gender, age-matched with the case group; ④ patient informed consent; ⑤complete personal information. All healthy controls underwent a comprehensive physical examination to exclude other factors that could impact the study. The study received approval from the institute’s Medical Ethics Committee.

### Clinical experiment data

The collected data include gender, age, BMI, and various laboratory indicators: white blood cell count (WBC), hemoglobin (Hb), platelet count (PLT), prothrombin time (PT), activated partial thromboplastin time (APTT), fibrinogen (FIB), D-dimer, alanine aminotransferase (ALT), aspartate aminotransferase (AST), alkaline phosphatase (ALP), lactate dehydrogenase (LDH), total cholesterol (TC), triglycerides (TG), high-density lipoprotein cholesterol (HDL-C), low-density lipoprotein cholesterol (LDL-C), serum apolipoprotein A1 (ApoA1), apolipoprotein B (ApoB), creatinine (Cr), and uric acid (UA).

### Definition group

This study evaluated the severity of bleeding in APL using the World Health Organization (WHO) severity rating system (**Supplementary Table S1**). APL patients were categorized into two groups according to their bleeding grade: individuals without significant bleeding (grades 0, 1, and 2) and those with significant bleeding (grades 3 and 4).

At the time of diagnosis, risk stratification was based on the WBC and PLT from the complete blood count (**Supplementary Table S2**). APL was classified into three risk groups: high, intermediate, and low. The risk stratification criteria are as follows: WBC <10×10^9^/L, PLT >40×10^9^/L indicate low risk, WBC <10×10^9^/L, PLT ≤40×10^9^/L indicate intermediate risk, and WBC ≥10×10^9^/L indicate high risk.

### Follow-up time

All patients received treatment according to the Chinese guidelines for acute promyelocytic leukemia, and the follow-up period was extended to one year from the onset of the disease. At the end of the one-year follow-up, patients were classified as either deceased or alive.

### Data processed

The data processing was conducted using SPSS version 27.0, while GraphPad Prism 10.0 was utilized to create charts. R Studio was employed to analyze the samples related to APL and hyperlipidemia. The Shapiro-Wilk test was performed to evaluate the normal distribution of the measurement data. Data following normal distribution were presented as mean ± standard deviation (X ± SD), while non-normally distributed data were presented as median (M) with quartiles (P25, P75). Count data were reported as the number of cases and percentages (%), with group comparisons conducted using the Chi-square test. Pearson correlation analysis assessed the correlation between blood lipids and APL. A significance level of P<0.05 was used to determine statistical significance.

### Downloaded hyperlipidemia and APL data

The APL datasets were obtained individually from the Gene Expression Omnibus (GEO) database at https://www.ncbi.nlm.nih.gov/geo/ (16). Specifically, the datasets GSE34577 (comprising 14 APL and 18 standard bone marrow samples) and GSE 1010 (consisting of 12 hyperlipidemia and 12 standard lipid samples) were utilized as the training set, whereas GSE 3059 (comprising 32 samples exhibiting abnormal lipid metabolism) served as the validation set.

### Differences in genetic analysis

The GEO2R (22) is a convenient online tool for analyzing differentially expressed genes (DEGs) in the Gene Expression Omnibus (GEO) comprehensive database. This study, GEO2R was used to identify significant differences in gene expression between APL patients and healthy control samples. Specifically, we analyzed gene expression data from the GSE34577 and GSE1010 datasets, screening out differentially expressed genes related to lipid metabolism (selection criteria: |log2FC| > 1, P < 0.05). These genes may be closely related to lipid metabolism disorders in APL and are potential targets for further research. By cross-analyzing DEGs from different datasets using R Studio, identifying co-expressed genes, and validating the expression status of these genes in an independent validation set, the reliability of the screening results is ensured. This step provides a clear gene candidate set to explore the mechanism of lipid metabolism in APL.

### The PPI network construction

In order to further elucidate the function and interactions of lipid metabolism-related genes in APL, we constructed a Protein-Protein Interaction (PPI) network using the online database STRING (https://string-db.org/cgi/input.pl) (23). STRING is a database that analyzes protein interactions, integrating experimental data, text mining, and prediction information. PPI network analysis can reveal potential collaborative mechanisms of proteins encoded by target genes in lipid metabolism and the pathogenesis of APL. The STRING database integrates experimental data and prediction information to help identify direct or indirect interactions of proteins related to lipid metabolism. Subsequently, the PPI network was visualized using Cytoscape 3.8 technology (24) a biological network visualization tool. Core genes in key hub positions in the network were identified by applying network topological parameters (such as node degree and betweenness centrality). Identifying key nodes in the network suggests that they may be involved in the pathogenesis and progression of APL through regulating lipid metabolism. The construction of the PPI network provides a foundation for elucidating the molecular functions of key genes.

### Functional enrichment analysis

In order to further explore the biological functions of the identified differentially expressed genes and the signaling pathways they participate in, we performed enrichment analysis on Gene Ontology (GO) and Kyoto Encyclopedia of Genes and Genomes (KEGG) pathways using the DAVID (http://www.david.abcc.ncifcrf.gov/) (25). GO analysis focused on the molecular functions of genes (such as lipid transport, regulation of lipid metabolism, etc.) and biological processes (such as cell proliferation and migration), while KEGG pathway analysis revealed significant enrichment of these genes in lipid metabolism-related pathways (such as the PPAR signaling pathway). These results further support the hypothesis that PTK2 may regulate the pathogenesis of APL through lipid metabolism-related pathways, providing a theoretical basis for subsequent functional validation.

### Cell culture

For the study of acute promyelocytic leukemia (APL), NB4 (ACC 207, DSMZ) and HL-60 (CCL-240™, ATCC) cell lines were chosen as models, while CD34+ cell line (PCS-800-012™, ATCC) was selected as a negative control. During culture, RPMI 1640 medium (Thermo Fisher Scientific, Cat No. 11875093) was used, supplemented with 10% fetal bovine serum (FBS, Thermo Fisher Scientific, Cat No. 10099141) and 1% penicillin-streptomycin (Penicillin-Streptomycin, Thermo Fisher Scientific, Cat No. 15140122) for NB4 cells, and 20% FBS and 1% penicillin-streptomycin for HL-60 cells. Cells were thawed rapidly in a 37°C water bath after cryopreservation, washed with RPMI 1640 medium to remove DMSO (Dimethyl Sulfoxide, DMSO, Sigma-Aldrich, Cat No. D4540), resuspended in culture medium, and seeded in T25 cell culture flasks (Corning, Cat No. 430639). The cells were then cultured in a 37°C, 5% CO₂ cell culture incubator for 24 hours for acclimation. Sub-culturing was performed when cell density reached 70-80%, involving washing, digestion, centrifugation, and reseeding in a fresh culture medium. Long-term storage of cells involved collection at appropriate densities, resuspending in a freezing medium containing 10% DMSO and 90% FBS (CryoStor, STEMCELL Technologies, Cat No. 3797), aliquoting into cryovials (Thermo Fisher Scientific, Cat No. 373857), gradual cooling, and transfer to liquid nitrogen storage.

### RNA extraction

Total RNA from NB4 and HL-60 cells was extracted using TRIzol reagent. Cells or tissue samples were lysed in TRIzol reagent under RNase-free conditions. After adding and mixing chloroform, centrifugation was carried out to separate phases, followed by precipitation of RNA with isopropanol, an ethanol wash to remove impurities, and final dissolution in DEPC-treated water. RNA quality and concentration were verified using Nanodrop or agarose gel electrophoresis.

### Reverse transcription

The extracted RNA was reverse-transcribed using the SuperScript IV First-Strand Synthesis System. Following the manufacturer’s protocol, the reaction included 1 µg of RNA, reverse transcriptase, random primers, dNTPs, and reaction buffer. The reaction was typically incubated at 42°C for 60 minutes, followed by heat inactivation at 75°C for 5 minutes. The synthesized cDNA was stored for subsequent qPCR analysis.

### qPCR

qPCR analysis was performed using SYBR Green PCR Master Mix to assess the expression levels of PTK2 and the reference gene (GAPDH). The qPCR cycling conditions were set according to the optimal annealing temperature for each primer, typically running 40 cycles of denaturation (95°C 15 seconds), annealing (60°C 30 seconds), and extension (72°C 30 seconds). Data analysis involved recording fluorescence signals, calculating relative expression levels based on Ct values, and utilizing the 2^-ΔΔ^Ct method.

### Western blot

Total protein from APL cells was extracted using RIPA buffer. Cells were lysed in RIPA buffer with protease inhibitors to prevent protein degradation. The lysate was clarified by centrifugation, and the protein concentration was quantified. SDS-PAGE separated protein samples, transferred to a PVDF membrane, and incubated with specific antibodies against PTK2 and the reference protein (GAPDH). After washing, secondary antibodies were applied, and protein bands were visualized using an ECL detection system. Protein expression was analyzed using image analysis software.

### Cell transfection

PTK2 gene was knocked down using small interfering RNA (siRNA) to study its function in APL cells (**Supplementary Figure S2**). Specific siRNAs targeting PTK2 were designed, and non-specific siRNA (siNC) was used as a control. Transfection was performed with Lipo3000 reagent. Cells were seeded in plates, and the transfection mixture was added to the medium for cell uptake. Transfected cells were cultured for 24-48 hours to allow for gene knockdown. PCR validated knockdown efficiency, and si PTK2#3 showed the highest silencing efficiency for subsequent experiments.

### ELISA

ELISA experiments were conducted to detect low-density lipoprotein (LDL) and fibrinogen (FIB) in NB4 and HL-60 cells. Cells were lysed after cell collection and stimulation, and LDL and FIB were extracted for detection using ELISA kits. Absorbance was measured using an ELISA reader, and concentrations were calculated from standard curves.

### CCK-8 assay

Cell proliferation was assessed using the CCK-8 assay kit. APL cells were seeded in plates, CCK-8 solution was added and incubated, and absorbance was measured to determine cell proliferation.

### Cell apoptosis

Cell apoptosis was detected using Annexin V-FITC/PI staining and flow cytometry. Stained cells were analyzed to determine the apoptosis rate based on fluorescence signals.

### Migration assay

Cell migration assay was performed using Transwell chambers to evaluate the impact of PTK2 on cell migration ability. Cells were seeded in the upper chamber and allowed to migrate to the lower chamber. After staining and fixation, migrated cells were counted under a microscope.

### Data analysis

Data analysis was conducted using SPSS 27.0 for statistical analysis and GraphPad Prism 10.0 for graphing. R Studio was used to analyze samples related to APL and hyperlipidemia. Shapiro-Wilk test was utilized for normality assessment. Data were presented as mean ± standard deviation for normally distributed data and median with quartiles for non-normally distributed data. The chi-square test was used for intergroup comparisons. Pearson correlation analysis evaluated the association between lipid levels and APL. Statistical significance was set at P<0.05.

## Results

### General information

This study included 90 newly diagnosed patients with APL and 99 healthy individuals who presented at our hospital during the same period (**Table 1**). In the APL group, 32 male patients constituted 35.2% of the sample, and 58 female patients represented 63.7% of the cohort. Among the healthy control group, 55 male participants comprised 55.6%, and 44 female participants accounted for 44.4%. The average ages of the healthy control group and the APL patient group were 48 ± 2.2 years and 49 ± 2.2 years, respectively, with no significant statistical difference (P = 0.488)(**Table 1**). Therefore, the differences in lipid metabolism indicators in this study are not influenced by age as a confounding factor.

**Table 1:** General information.

Clinical Characteristics	APL	Control	P
Year	49 ± 2.2	48 ± 2.2	0.488
Sex (N)			0.006*
Male	32	55	
Female	58	44	
BMI (Kg/m2)	24.46	23.2	0.024*
PT (s)	12.7 (11.8,14.8)	12.4 (11.9,13.0)	<0.001*
APTT (s)	27.0 (23.3,31.2)	31.7 (28.9,34.3)	<0.001*
D-Dimer (mg/L)	4.04 (3.06,14.65)	0.71 (0.55,1.51)	<0.001*
FIB (g/L)	1.71 (1.06,2.48)	2.85 (2.40,3.60)	<0.001*
WBC (10*9/L)	1.55 (0.90,4.35)	5.89 (4.78, 7.33)	<0.001*
Hb (g/L)	83.5 (64.8,101.0)	138 (120,150)	<0.001*
PLT (10*9/L)	26.1 (13.8.40.5)	224.0 (186.5,270.0)	<0.001*
ALT (U/L)	21 (15,38)	17 (12,24)	<0.001*
AST (U/L)	23 (17,33)	19 (16,24)	<0.001*
ALP (U/L)	80 (67,99)	73 (61,98)	0.308
LDH (U/L)	254 (163,373)	162 (141,182)	<0.001*
TC (mmol/L)	4.26 (3.91,4.87)	4.33 (3.77,5.07)	0.264
TG (mmol/L)	1.93 (1.13,2.95)	1.15 (0.77,1.47)	<0.001*
HDL-C (mmol/L)	0.95 (0.86, 1.27)	1.18 (1.06,1.37)	<0.001*
Apo A1 (g/L)	1.07 (0.94,1.32)	1.20 (1.07,1.38)	0.045*
ApoB (g/L)	0.90 (0.77,0.99)	0.87 (0.71,1.00)	0.225
CR (μmol/L)	60.0 (48.8,69.8)	55.0 (18.0,69.1)	0.031*
LDL-C (mmol/L)	2.34 ± 0.60	2.53 ± 0.62	0.028*
UA (μmol/L)	250.25 ± 85.68	282.5 ± 92.325	0.014*

WBC, white blood cell; Hb, hemoglobin; PLT, platelets; PT, Prothrombin time; APTT, Partial prothrombin time; FIB, fibrinogen; ALT, Alanine aminotransferase; AST, Aspartate aminotransferase; ALP, Alkaline phosphatase; CR, Creatinine; UA, Uric acid; LDH, Lactate dehydrogenase; TC, Total cholesterol; TG, Triglyceride; HDL-C, High density lipoprotein cholesterol; LDL-C, Low density lipoprotein cholesterol; Apo A1, Apolipoprotein A1; ApoB, Apolipoprotein B; *P<0.05.

Bold values indicates significant p<0.05 results.

The results showed that in APL patients, the levels of lactate dehydrogenase (LDH) and triglycerides (TG) were significantly higher than those in the control group (P < 0.001)(**Table 1**). Conversely, HDL, APOA1, and LDL levels were significantly reduced in the APL group compared to the controls (P < 0.05)(**Table 1**). However, the two groups had no significant differences in APOB and TC levels (P > 0.05)(**Table 1**). Furthermore, WBC, Hb, PLT, and other indices were notably lower in patients with APL compared to the control group (P < 0.05)(**Table 1**). Significant variations were also observed in PT, APTT, D-dimer, and fibrinogen levels (P < 0.05)(**Table 1**). Although ALT, AST, CR, and CK displayed statistically significant differences between the APL and control groups (P < 0.05)(**Table 1**), their median values fell within the normal reference ranges, indicating no significant clinical implications.

### LDH and TG increase the risk of APL

The research results indicate that the elevation of lactate dehydrogenase (LDH) and triglyceride (TG) levels is significantly associated with the risk of acute promyelocytic leukemia (APL) (26, 27). The univariate logistic regression analysis results (**Supplementary Table S3**) indicated that factors associated with APL included LDH (Wald = 33.64, P = 0.000), TG (Wald = 24.69, P = 0.000), HDL-C (Wald = 6.45, P = 0.01), and LDL-C (Wald = 4.71, P = 0.03). However, no significant correlation was found between TC (P = 0.55), APOA1 (P = 0.13), and APL (**Supplementary Table S3**). Subsequent analysis revealed that gender (Wald = 7.48, P = 0.006), BMI (Wald = 7.30, P = 0.007), ALT (Wald = 11.04, P = 0.00), AST (Wald = 12.33, P = 0.00), Cr (Wald = 5.59, P = 0.02), and UA (Wald = 5.84, P = 0.02) were correlated with APL (**Supplementary Table S3**).

Following univariate analysis, a logistic regression model was employed to perform a multifactor analysis of serum lipid and LDH levels. The detailed results are presented in **Table 2**. In the adjusted results without confounding variables (Model 1), LDH and TC exhibited a positive correlation with the incidence of APL. After adjusting for gender and BMI in Model 2, the risk of APL increased by 2.4% per unit increase in LDH (OR=1.024, 95% CI: 1.01-1.03) and by 80.1% per unit increase in TG (OR=1.081, 95% CI: 1.09-2.99). After further adjustment for AST, ALT, and UA in Model 3, the likelihood of APL rose by 2.3% per unit rise in LDH (OR=1.023, 95% CI: 1.01-1.03) and by 1.113 times per unit increase in LDL (OR=2.113, 95% CI: 1.13-3.95). LDH and TG emerged as independent risk factors associated with an increased APL risk.

**Table 2:** The correlations between serum lipid index indicators and APL using multivariate logistic regression analysis.

	Model 1		Model 2		Model 3	
	P	OR (95% CI)	P	OR (95% CI)	P	OR (95% CI)
LDH	0.001^*^	1.024 (1.02,1.03)	0.000^*^	1.024 (1.01,1.03)	0.000^*^	1.023 (1.01,1.03)
TG	0.013^*^	1.848 (1.14,2.99)	0.023^*^	1.801 (1.09,2.99)	0.019^*^	2.113 (1.13,3.95)
HDL-C	0.876	0.979 (0.76,1.27)	0.837	0.869 (0.23,3.30)	0.892	0.908 (0.22,3.68)
LDL-C	0.15	0.625 (0.33,1.19)	0.223	0.662 (0.34,1.29)	0.111	0.55 (0.26, 1.15)

Model 1, crude model.

Model 2, adjusted for BMI, Sex.

Model 3, adjusted for BMI, Sex, Cr, UA, ALT, AST.

LDH, Lactate dehydrogenase; TG, Triglyceride; HDL-C, High density lipoprotein cholesterol; LDL-C, Low density lipoprotein cholesterol; CI, Confidence interval.

*P<0.05.

Bold values indicates significant p<0.05 results.

### TG and HDL-C associated with the risk stratification of APL

**Figure 1:**
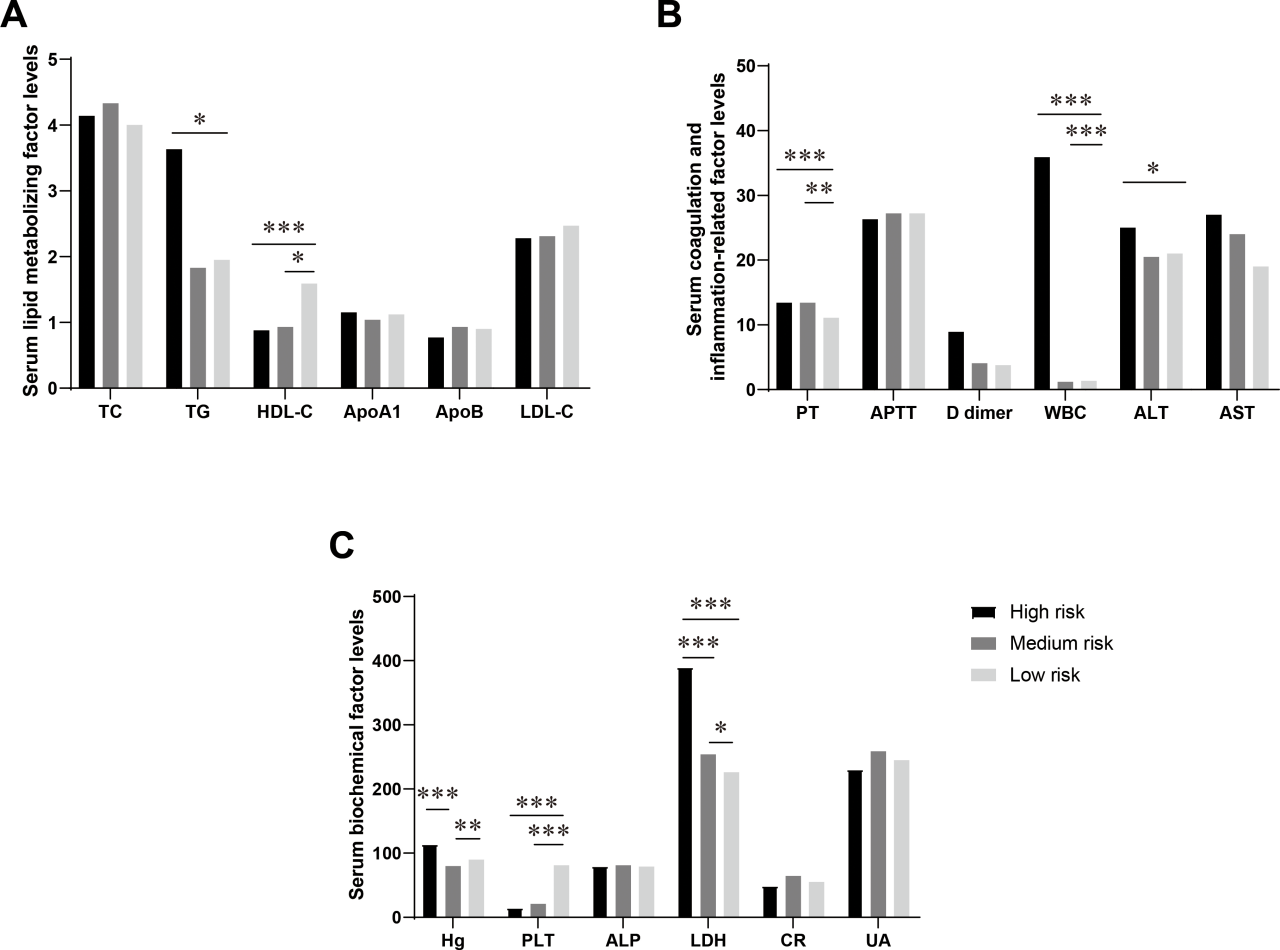
Differences in the levels of serum lipid metabolism-related factors in APL patients in different risk-stratified groups. **(A)** Comparison of serum lipid metabolism factors levels in APL patients in different risk-stratified groups. This figure shows the levels of serum total cholesterol (TC, mmol/L), triglycerides (TG, mmol/L), high-density lipoprotein cholesterol (HDL-C, mmol/L), low-density lipoprotein cholesterol (LDL-C, mmol/L), apolipoprotein A1 (ApoA1, g/L), and apolipoprotein B (ApoB, g/L) in patients with acute promyelocytic leukemia (APL) in the high-risk group, intermediate-risk group, and low-risk group. **(B)** Comparison of levels of serum coagulation and inflammation-related factors in APL patients in different risk-stratified groups. This figure shows the levels of plasma prothrombin time (PT, s), activated partial thromboplastin time (APTT, s), D-dimer (mg/L), white blood cells (WBC, 10^9^/L), alanine aminotransferase (ALT, U/L), and aspartate aminotransferase (AST, U/L) in patients with APL in the high-risk group, intermediate-risk group, and low-risk group. **(C)** Comparison of levels of serum biochemical factors in APL patients in different risk-stratified groups. This figure shows the levels of hemoglobin (Hb, g/L), platelets (PLT, 10^9^/L), alkaline phosphatase (ALP, U/L), lactate dehydrogenase (LDH, U/L), creatinine (Cr, mmol/L), and uric acid (UA, mmol/L) in patients with APL in the high-risk group, intermediate-risk group, and low-risk group. Data are presented as mean ± standard deviation or median (interquartile range), and differences between groups were compared using one-way analysis of variance or Kruskal-Wallis test, *P<0.05, **P<0.01, ***P<0.0001.

Risk stratification of acute promyelocytic leukemia (APL) is significantly associated with various metabolic indicators, and this finding holds important clinical significance for early risk prediction of APL (26, 27). In a cohort of 90 APL patients, 18 individuals (19.8%) were categorized as high risk, 53 patients (58.2%) as medium risk, and 19 patients (20.9%) as low risk (**Supplementary Table S4**). The levels of LDH showed a progressive rise according to the risk stratification in various APL classifications, with a statistically significant variation observed among the three risk groups (P < 0.001) (**Figure 1**). Concurrently, there was a gradual decline in HDL-C levels as the risk level increased, with the most notable difference observed between the low-risk and medium-high-risk groups (P < 0.001) (**Figure 1**). TG levels were elevated in the high-risk category and decreased in the medium-low-risk category, with a statistically significant contrast noted between the low-risk and high-risk groups (P < 0.05) (**Figure 1**). The FIB level increased with a higher risk level, demonstrating a statistically significant difference between the low-risk and high-risk groups. Additionally, the PT value exhibited a prolonged elevation in the middle and high-risk groups, with a notably significant difference from the low-risk group (P < 0.05) (**Figure 1**). No significant differences among risk-stratified APL categories were found in serum lipid indexes, including APOA1, APOB, LDL, and TC. Furthermore, no significant differences among the groups were observed in Cr, AST, and ALT levels (P > 0.05) (**Figure 1**).

PT, FIB, ALT, HDL-C, and TG were incorporated into the multiple-ordered logistic regression model, and the corresponding outcomes are presented in **Table 3** below. The TG level directly correlated with APL risk classification, whereas HDL showed an inverse relationship with APL risk stratification. Therefore, increased TG and decreased HDL levels were associated with higher APL risk stratification.

**Table 3:** Exploring the associations between indicators of serum lipid index and different risk stratification APL using multivariate logistic regression.

	B	STD	Wals	P	95% CI
LDH	0.002	0.002	1.57	0.21	(-0.001, 0.006)
TG	0.284	0.134	4.472	0.034^*^	(0.021, 0.548)
HDL-C	2.087	0.722	8.347	0.004^*^	(-3.503, -0.671)
PT	0.148	0.11	1.816	0.178	(-0.067 - 0.363)
FIB	0.413	0.194	4.522	0.033	(-0.794, -0.032)
ALT	0.013	0.01	1.883	0.17	(-0.006, 0.032)

LDH, Lactate dehydrogenase; TG, Triglyceride; HDL-C, High-density lipoprotein cholesterol; PT, Prothrombin time; FIB, fibrinogen; ALT, Alanine aminotransferase.

*P<0.05.

Bold values indicates significant p<0.05 results.

### Lipid levels were lower in people at higher blood risk

**Figure 2:**
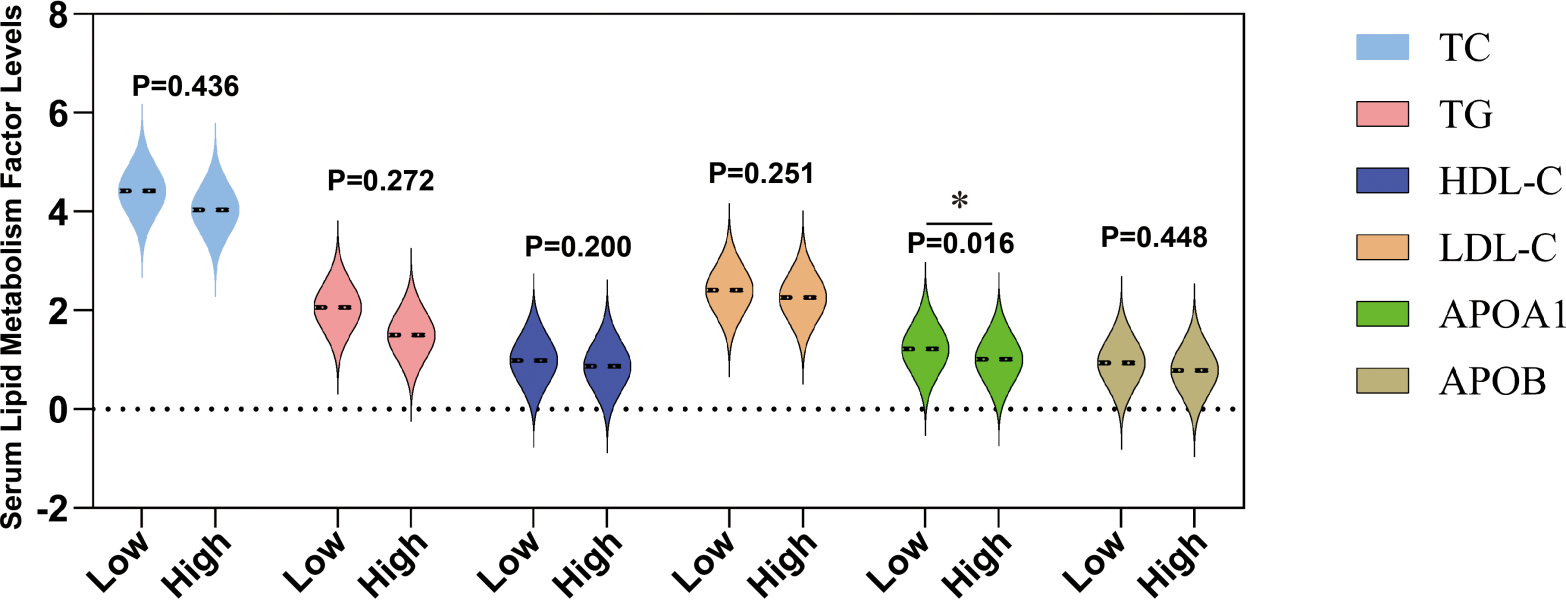
Differences in the levels of serum lipids at different bleeding risk stratifications. This figure shows the levels of serum total cholesterol (TC, mmol/L), triglycerides (TG, mmol/L), high-density lipoprotein cholesterol (HDL-C, mmol/L), low-density lipoprotein cholesterol (LDL-C, mmol/L), apolipoprotein A1 (ApoA1, g/L), and apolipoprotein B (ApoB, g/L) in patients classified into low bleeding risk group (low) and high bleeding risk group (high). *P < 0.05.

In acute promyelocytic leukemia (APL), changes in lipid metabolism are closely related to the risk of bleeding, especially in patients at high risk of hemorrhage. The significant reduction in serum lipid levels in high-risk blood patients provides a basis for further exploration of its potential mechanism (28, 29). **Supplementary Table S5** shows comprehensive statistical data of all patients (including males and females) categorized as having a high risk of bleeding APL according to the WHO grading criteria. Specifically, compared to 47 low-risk bleeding APL patients, the levels of apolipoprotein A1 (APOA1) in 43 high-risk bleeding patients were significantly reduced (P=0.016) (**Figure 2**), indicating that metabolic abnormalities may play a crucial role in bleeding risk. Although LDH showed a rising trend in the high-risk blood group, it did not reach a significant difference (**Figure 2**). In addition, other lipid indicators such as total cholesterol (TC), triglycerides (TG), low-density lipoprotein (LDL), and high-density lipoprotein (HDL) showed no statistical differences between the high and low-risk groups (**Figure 2**). These results further suggest that changes in blood lipid levels in high-risk APL patients are closely related to the increased risk of bleeding, especially the possible association between low LDL-C levels and higher bleeding risk. These findings help identify high-risk bleeding patients early in clinical practice and improve the treatment management strategy for APL.

### LDL associated with poor prognosis in APL

Low-density lipoprotein (LDL) is not only associated with cardiovascular disease, but recent studies have shown that it also plays a role in tumor progression and inflammation. In acute promyelocytic leukemia (APL), changes in LDL levels may be associated with prognosis through their effects on inflammation and cell apoptosis (30). Our study employed univariate and multivariate regression analyses to investigate the association between several biomarkers and Acute Promyelocytic Leukemia (APL) prognosis. The results revealed significant associations between APL prognosis and levels of PT (P = 0.01), FIB (P = 0.004), LDH (P = 0.003), and LDL [P = 0.038 (**Table 4**)]. Subsequent multiple logistic regression models demonstrated that FIB and LDL were independent risk factors, exerting a notable influence on the prognosis of APL. Specifically, each additional unit increase in FIB levels was associated with a substantial 91.4% decrease in the risk of death for APL patients (**Table 4**). Similarly, an increase of one unit in LDL levels corresponded to an 82% lower risk of mortality among individuals with APL (**Table 4**). This suggests that LDL and FIB may have potential clinical value as independent biomarkers in the prognostic assessment of APL patients.

**Table 4:** Univariate and multi-factor analysis of prognostic correlation in APL.

Univariate analysis			Multivariate analysis	
	P	OR (95% CI)	P	OR (95% CI)
Year	0.096	1.037 (0.994, 1.082)		
Gender	0.756	1.238 (0.322, 4.757)		
BMI (Kg/m2)	0.772	0.97 (0.791, 1.19)		
PT (s)	0.01^*^	1.315 (1.068, 1.618)	0.344	1.161 (0.852, 1.583)
APTT (s)	0.978	1.001 (0.917, 1.093)		
D-Dimer (mg/L)	0.396	1.023 (0.971, 1.077)		
FIB (g/L)	0.004^*^	0.059 (0.009, 0.409)	0.031^*^	0.086 (0.009, 0.802)
WBC (10*9/L)	0.838	1.003 (0.979, 1.027)		
Hb(g/L)	0.511	0.991 (0.965, 1.018)		
PLT (10*9/L)	0.659	0.996 (0.977, 1.015)		
ALT (U/L)	0.578	0.992 (0.965, 1.02)		
AST (U/L)	0.712	1.004 (0.982, 1.026)		
ALP (U/L)	0.718	0.996 (0.972, 1.02)		
LDH (U/L)	0.003^*^	1.004 (1.002, 1.007)	0.535	1.001 (0.997, 1.005)
TC (mmol/L)	0.687	0.882 (0.477, 1.627)		
TG (mmol/L)	0.836	0.965 (0.69, 1.35)		
HDL-C (mmol/L)	0.389	0.425 (0.061, 2.972)		
LDL-C (mmol/L)	0.038^*^	0.276 (0.081, 0.934)	0.031^*^	0.18 (0.038, 0.858)
Apo A1(g/L)	0.726	0.718 (0.112, 4.58)		
ApoB (g/L)	0.197	2.95 (0.57, 15.262)		
Cr (mmol/L)	0.621	1.004 (0.988, 1.02)		
UA (mmol/L)	0.324	1.003 (0.997, 1.01)		

WBC, white blood cell; Hb, hemoglobin; PLT, platelets; PT, Prothrombin time; APTT, Partial prothrombin time; FIB, fibrinogen; ALT, Alanine aminotransferase; AST, Aspartate aminotransferase; ALP, Alkaline phosphatase; CR, Creatinine; UA, Uric acid; LDH, Lactate dehydrogenase; TC, Total cholesterol; TG, Triglyceride; HDL-C, High-density lipoprotein cholesterol; LDL-C, Low-density lipoprotein cholesterol; Apo A1, Apolipoprotein A1; ApoB, Apolipoprotein B. *P<0.05.

Bold values indicates significant p<0.05 results.

### No association between APL survival and LDH or lipids

**Figure 3:**
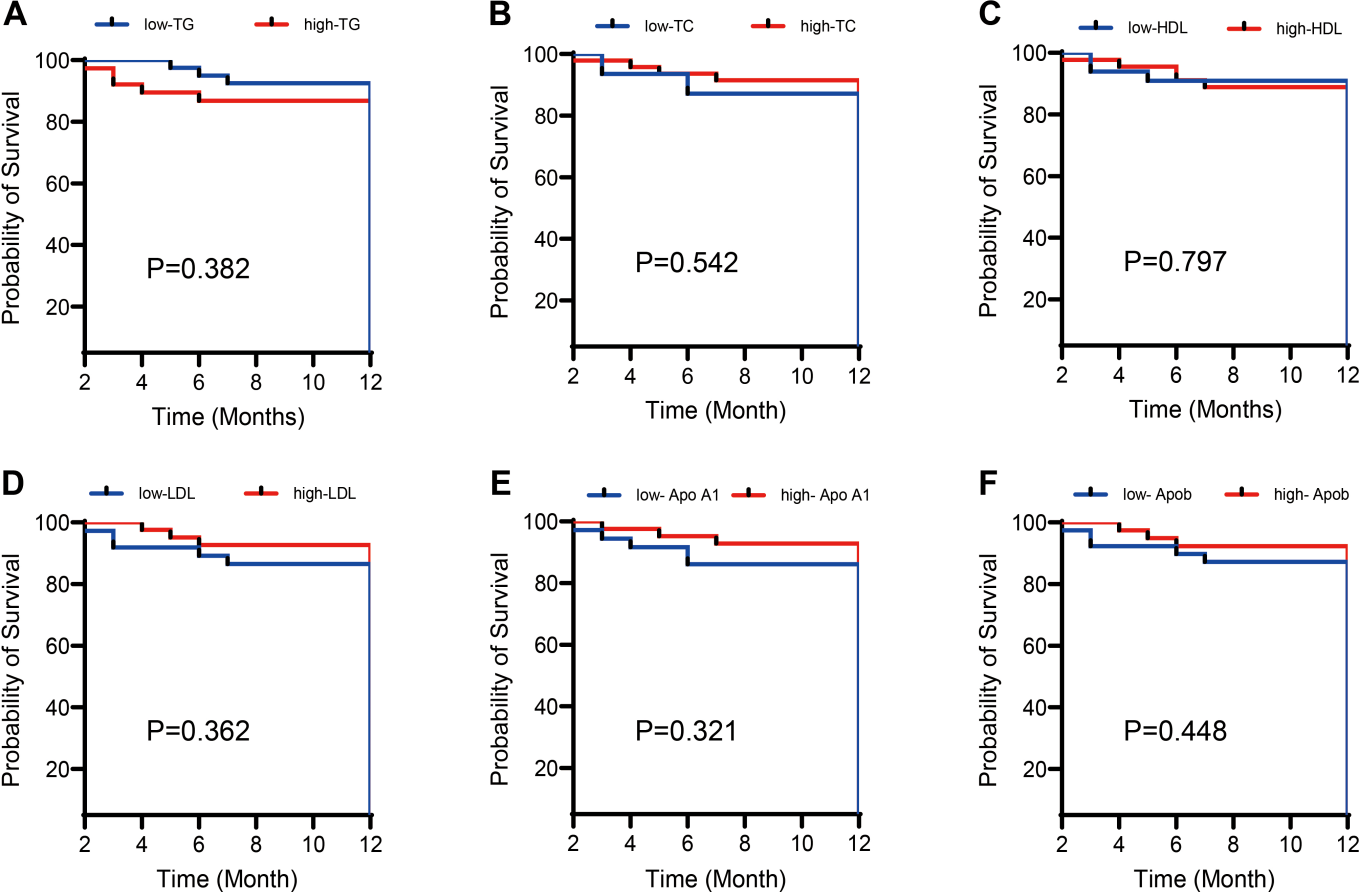
Effects of different lipid levels and LDH levels on the survival rate of APL patients. Kaplan-Meier survival analysis compared the impact of different lipid levels (including **(A)** Total cholesterol (TC), **(B)** Triglycerides (TG), **(C)** High-density lipoprotein cholesterol (HDL-C), **(D)** Low-density lipoprotein cholesterol (LDL-C), **(E)** Apolipoprotein A1 (ApoA1), **(F)** Apolipoprotein B (ApoB)) and Lactate dehydrogenase (LDH) levels on the survival rate of patients with acute promyelocytic leukemia (APL). The results show that neither lipid nor LDH levels significantly impact the survival rate of APL patients (P > 0.05).

The APL patients were stratified into higher and lower groups based on the median TC, TG, HDL-C, ApoA1, ApoB, and LDL-C values. Kaplan-Meier analysis was conducted, with the results in **Figure 3** revealing that neither blood lipid levels nor LDH had a statistically significant impact on APL survival (P > 0.05).

### Differential expression, interaction network, and functional enrichment analysis of PTK2

**Figure 4:**
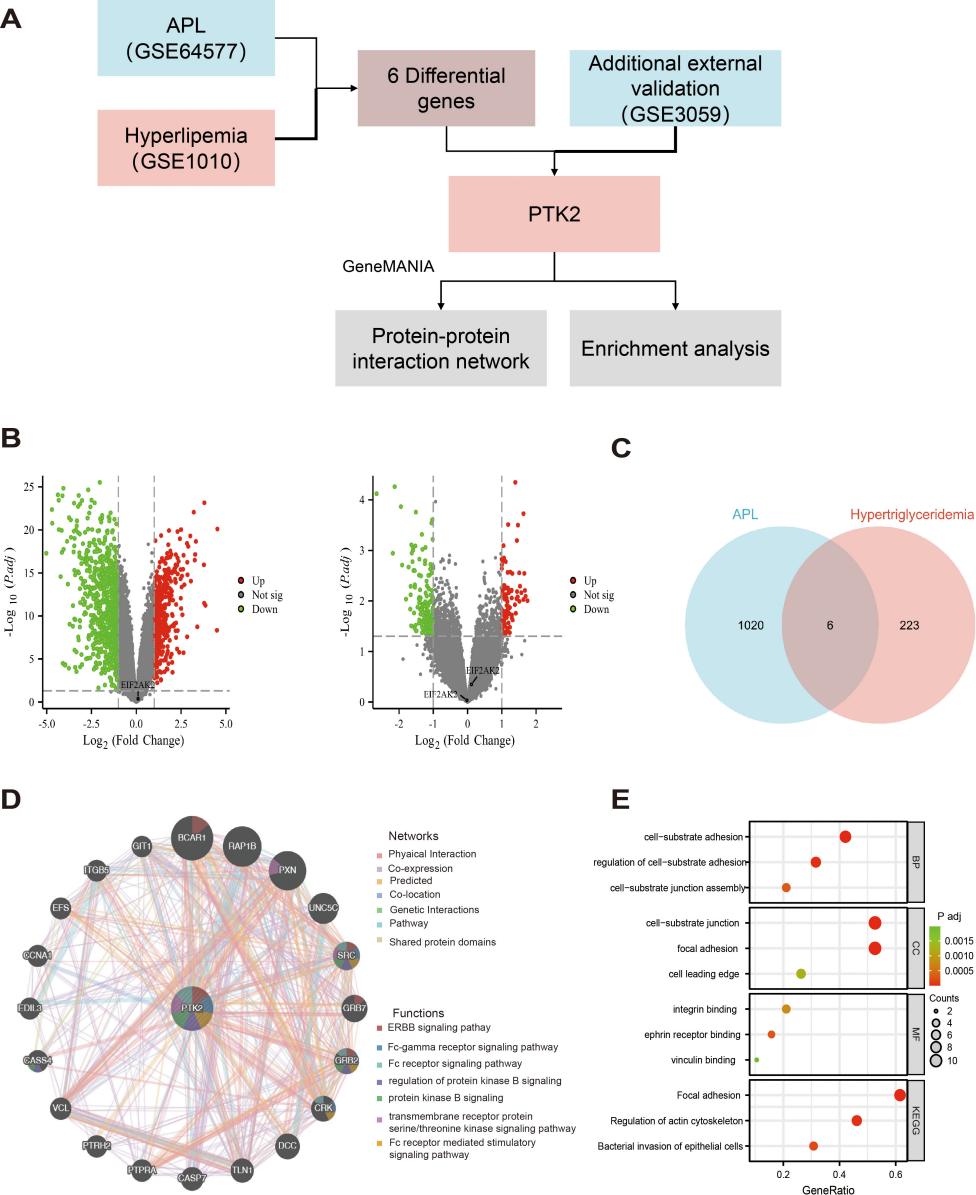
Differential gene expression screening, PTK2 interaction network, and functional enrichment analysis. **(A)** The research flowchart demonstrates the process of selecting differentially expressed genes from the GSE64577 and GSE1010 datasets, ultimately confirming PTK2 as a key gene. **(B)** (Left) Volcano plot of up-regulated and down-regulated genes in the GSE64577 dataset; (Right) Volcano plot of up-regulated and down-regulated genes in the GSE1010 dataset. Red represents up-regulated genes, and green represents down-regulated genes. **(C)** Six differentially expressed genes were selected through strict criteria (P value <0.05 and |log2FC|>1). **(D)** In GeneMANIA analysis, the top 20 proteins were predicted to interact with PTK2 and their interaction networks, with corresponding networks and functions marked in different colors. **(E)** Functional and pathway enrichment analysis results of PTK2 and its interacting protein genes. The redder the color, the lower the p-value; the greener the color, the higher. The size of the circle represents the proportion of genes.

In this study, we first screened differentially expressed genes by analyzing the GSE64577 and GSE1010 datasets and ultimately confirmed PTK2 as a key gene. The research flowchart in **Figure 4A** shows the process of screening differentially expressed gene PTK2 from the dataset, including using GeneMANIA for interaction network analysis and enrichment analysis.

In the GSE64577 dataset (left side of **Figure 4B**), 404 genes were upregulated, and 871 genes were downregulated, while in the GSE1010 dataset (right side of **Figure 4B**), 89 genes were upregulated and 164 genes were downregulated. By applying stringent criteria (P value <0.05 and |log2FC|>1), 6 differentially expressed genes were selected in the dataset, as shown in **Figure 4C**. Subsequently, revalidation in the external dataset GSE3059 confirmed PTK2 as the final differentially expressed gene.

GeneMANIA analysis identified the top 20 predicted interacting proteins with PTK2, including BCAR1, RAP1B, PXN, UNC5C, SRC, GRB7, GRB2, CRK, DCC, TLN1, CASP7, PTPRA, PTRH2, VCL, CASS4, EDIL3, CCNA1, EFS, ITGB5, and GIT1, as shown in **Figure 4D**. Network analysis showed that 70.9% of the proteins involved physical interactions, and 16.01% were co-expressed proteins. In addition, 4.96% were predicted interactions, 3.22% were involved in co-localization, 2.63% had gene-level interaction correlations, 1.74% were associated with pathways, and 0.55% shared protein domains. Furthermore, genes related to PTK2 may exhibit standard biological functions and pathways. Further functional analysis using GeneMANIA revealed that PTK2, SRC, and GRB2 can interact in multiple cellular signaling pathways, including the ERBB signaling pathway, FC receptor signaling pathway, and Fc receptor-mediated stimulation signaling pathways. They also regulate protein kinase B signaling and transmembrane receptor protein tyrosine/threonine kinase signaling. PTK2 and CRK are involved in the protein kinase B signaling pathway, FC receptor signaling pathway, and Fc receptor-mediated stimulation signaling pathway. PTK2, BCAR1, and GRB7 are related to the ERBB signaling pathway, while PXN is associated with the tyrosine/threonine kinase signaling pathway.

To enhance our understanding of the functional role of PTK2, we conducted functional and pathway enrichment analysis of PTK2 and its interacting protein genes (**Figure 4E**). In biological processes, focal adhesions play a core role in cell-matrix adhesion, regulation of cell-matrix adhesion, and assembly of cell-matrix connections. In cellular structures, focal adhesions primarily localize to cell-matrix connections, adhesion plaques, and the leading edge of cells. Regarding molecular functions, focal adhesions mainly bind to integrins, adrenergic receptors, and actin. Analysis from the KEGG database shows significant enrichment of biological functions related to adhesion plaques and regulation of actin cytoskeleton in the cell.

### The role of PTK2 in the proliferation, migration, and lipid metabolism of APL cells

**Figure 5:**
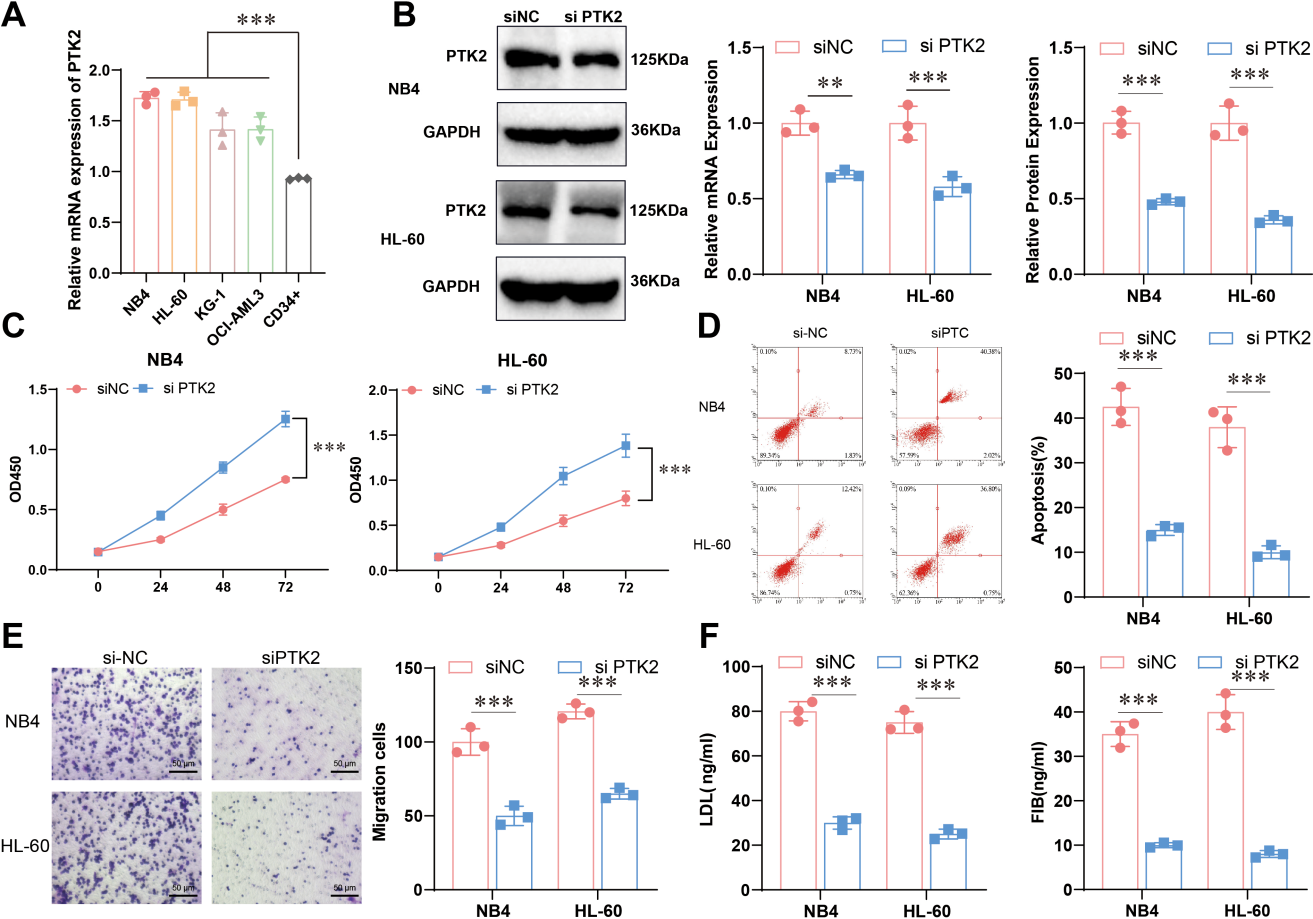
Expression and functional experiments of PTK2 in APL. **(A)** The mRNA expression levels of PTK2 in different cells were detected by qRT-PCR, n=3, t-test analysis, *** represents p<0.001. **(B)** Construction of the PTK2 knockout model was confirmed by Western blot and qRT-PCR, n=3. Red represents NB4, blue represents HL-60, t-test analysis, ** represents p<0.01, *** represents p<0.001; **(C)** Cell proliferation capacity was evaluated by CCK-8 experiment, n=5, two-way ANOVA analysis, *** represents p<0.001. **(D)** Cell apoptosis rate was detected by Annexin V-FITC/PI staining method, n=3, t-test analysis, *** represents p<0.001. **(E)** Cell migration ability was evaluated by Transwell experiment, n=3, t-test analysis, *** represents p<0.001. **(F)** ELISA, n=5, detected levels of LDL and FIB. The statistical method used a t-test; P < 0.05 was statistically significant, and *** represents p<0.001.

Based on the results of bioinformatics analysis, to further explore the specific functions of PTK2 in acute promyelocytic leukemia (APL), we conducted cell experiments to validate further its role in cell proliferation, migration, and lipid metabolism. The experimental results showed that the expression of PTK2 in multiple cell lines was significant, with the expression levels of PTK2 in APL (NB4 and HL-60) significantly higher than the control group (CD34^+^), indicating that PTK2 plays an important role in the occurrence and development of APL (**Figure 5A**). We ultimately chose NB4 and HL-60 for further study based on these results. To further investigate the function of PTK2, we constructed a cell model with PTK2 knockdown, and the results showed a significant decrease in PTK2 expression in the knockdown group (**Figure 5B**). Cell proliferation ability assessed by the CCK-8 experiment showed a significant decrease in cell proliferation ability with PTK2 knockout (P < 0.01) (**Figure 5C**). In addition, results from Annexin V-FITC/PI staining showed that the apoptosis rate of the knockout group cells was significantly higher than that of the control group (P < 0.01), indicating that PTK2 knockout significantly increased cell apoptosis (**Figure 5D**). In migration experiments, the overexpression of PTK2 significantly promoted the migration ability of NB4 and HL-60 cells, while PTK2 knockout significantly inhibited cell migration ability (**Figure 5E**). Further, ELISA results indicated that the overexpression of PTK2 significantly increased the levels of low-density lipoprotein (LDL) and fibrinogen (FIB) in NB4 and HL-60 cells (P < 0.01), while in the PTK2 knockout group, the expression levels of LDL and FIB were significantly decreased (P < 0.01) (**Figure 5F**). These results demonstrate that PTK2 plays an important biological role in APL cells by regulating lipid metabolism and promoting cell proliferation and migration.

## Discussion

This study analyzed the serum lipid levels of patients with acute promyelocytic leukemia (APL) and healthy controls, showing significant lipid metabolism abnormalities in APL patients, characterized by elevated triglycerides (TG) and decreased levels of high-density lipoprotein cholesterol (HDL-C) and low-density lipoprotein cholesterol (LDL-C). Previous studies (31) have also mentioned lipid metabolism abnormalities in APL patients but only pointed out that the increase in TG levels may be related to the pathogenesis of APL, especially in connection with lipid metabolism disorders during ATRA therapy (13). This study also found that the PTK2 gene may play a significant role in APL by regulating lipid metabolism, cell proliferation, and migration processes. Furthermore, serum lipid levels may have potential indicative significance in the pathogenesis and risk stratification of APL patients, suggesting that the PTK2 gene could become a new therapeutic target, providing a new perspective for understanding the pathogenesis of APL.

Several studies have shown lipid metabolism reprogramming in various malignant tumors, with significant upregulation of lipid synthesis in solid tumors and abnormal activation of lipid synthesis pathways in blood tumors (32). Adipocytes have adverse regulatory effects in the bone marrow microenvironment, as they can surround tumor tissue to evade destruction and secrete metabolic regulators that affect the energy metabolism and insulin sensitivity of surrounding cells, thereby modulating the bone marrow microenvironment, inhibiting normal hematopoietic stem cell function, and promoting leukemia cell proliferation and differentiation (33, 34). Research indicates that changes in adipocyte metabolism in close contact with leukemia cells involve increased phosphorylation of adipose lipase and accelerated lipolysis, transferring fatty acids to meet the nutritional needs of leukemia cells. Molecular mechanism studies have found high fatty acid-binding protein FABP4 expression in leukemia cells, which competitively consume fatty acids. Knocking down FABP4 *in vitro* or using small-molecule inhibitors significantly inhibits leukemia cell proliferation, and downregulating FABP4 can inhibit leukemia progression and extend mouse survival (35). Adipocytes influence leukemia cell differentiation and survival and the cytotoxic effects of radiotherapy and chemotherapy drugs. When adipocytes are present, leukemia cells exhibit reduced responsiveness to doxorubicin, leading to increased survival rates due to the absorption of chemotherapeutic agents by adipocytes, lowering their concentration around leukemia cells and altering the molecular structure of chemotherapeutic drugs to render them inactive or induce leukemia cells to be unresponsive, thereby reducing their cytotoxicity (36). This study focuses on changes in lipid components such as HDL-C and LDL-C, revealing the association of elevated TG levels with high-risk APL patients, which is consistent with previous research results in acute myeloid leukemia (AML) and other cancers (37). It indicates that lipid metabolism abnormalities significantly affect APL development and progression. Moreover, the study finds a potential link between high triglyceride levels and the PPAR signaling pathway, suggesting that monitoring lipid metabolism abnormalities as indicators for risk stratification in APL progression is clinically applicable and feasible. Future research can further explore the relationship between the fatty acid-binding protein FABP4 and this pathway.

APOA1 plays a key role in high-density lipoprotein transport, exhibiting anti-inflammatory and anti-apoptotic characteristics (38). It may also indirectly affect coagulation processes by improving vascular health and blood circulation (39, 40). Previous studies have shown the significant cytotoxic effects of oxidized low-density lipoprotein (ox-LDL), impacting cell function and closely linked to cardiovascular events and inflammatory reactions in hematological diseases (41, 42). Given this, further investigation into the role of LDL-C in bleeding risk in APL is essential. This study found that low levels of APOA1 are associated with bleeding risk, while low levels of LDL-C are associated with poor prognosis. This suggests that lipid metabolism abnormalities affect the onset of APL and involve complications and prognosis. Changes in blood lipid levels can reflect the disease state and risk of complications in APL patients, providing theoretical support for lipid metabolism as a potential biomarker for bleeding tendency in APL patients. Additionally, the study reveals that patients at high risk of bleeding have significantly lower Hb levels than those at low risk (65 vs. 93 g/L, P < 0.001). The decrease in Hb levels may reflect bleeding severity and bone marrow hematopoietic function inhibition. Low Hb levels are important indicators of deteriorating conditions in APL patients, notably associated with bleeding risks. Furthermore, since there are gender differences in normal Hb levels between males and females, the Hb data in the study are comprehensive statistical results for all patients. Future studies may further analyze stratified data by gender to more comprehensively assess the impact of gender on anemia and bleeding risks.

In recent years, there have been significant advances in LDH therapy research for solid tumors, as inhibiting LDH can improve the tumor microenvironment and weaken the immunosuppressive cell action (43, 44). Changes in serum lipid markers contribute to risk stratification in APL, with TG and HDL-C levels particularly critical. Moreover, a significant increase in LDH levels could provide references for the diagnosis and prognosis of APL, as it has already been confirmed as an adverse prognostic indicator in solid tumors (14). The findings of this study pave the way for new clinical applications in the early diagnosis and personalized treatment of APL, revealing the unique significance of lipid profiles in risk stratification and prognosis of APL. Therefore, it is recommended to introduce lipid metabolism and LDH monitoring in APL patient management and consider the PTK2 gene as a potential target gene for developing more precise APL treatment strategies.

However, this study has its limitations. Firstly, the sample size is small, with only 90 cases of APL patients, leading to insufficient representativeness of the sample. The data is derived from a single-center study with a short follow-up time and no consideration of other treatments’ potential mixed effects on lipid metabolism, potentially resulting in selection bias. Subsequent research should involve multicenter data to enhance generalizability. Secondly, analyzing the expression of PTK2 using various methods has certain limitations in sensitivity and specificity. Thirdly, although technologies like CRISPR-Cas9 have been widely used in malignant tumor research, more gene editing methods should be employed in future explorations of the regulatory roles of LDH and PTK2 genes to delve deeper into their molecular mechanisms in APL. Additionally, this study has unresolved issues, such as the direct relationship between lipid metabolism abnormalities and APL relapse. Future studies should increase the sample size and conduct long-term follow-ups to investigate the relationship between lipid metabolism and disease recurrence. The specific mechanisms of the PTK2 gene in the lipid metabolism pathway require further research, which can be accomplished by combining transcriptome and metabolome data to dissect the regulatory role of PTK2 in the complex metabolic network. PCSK9 inhibitors have shown progress in lipid metabolism applications and could be explored for their potential in APL treatment, aiding in designing more precise treatment strategies.

In conclusion, this study reveals the changes in serum lipid levels in newly diagnosed APL patients and their association with PTK2 gene expression, expanding the understanding of the role of lipid metabolism disorders in the pathogenesis of APL. Scientifically, it provides a new perspective for exploring the molecular mechanisms of APL; clinically, serum lipid markers and LDH levels are likely to be used for early diagnosis and prognosis evaluation in APL patients, while the PTK2 gene holds promise as a new therapeutic target.

## Conclusion

This study not only revealed significant lipid metabolism abnormalities in newly diagnosed acute promyelocytic leukemia (APL) patients, characterized by elevated triglycerides (TG) and decreased high-density lipoprotein cholesterol (HDL-C), low-density lipoprotein cholesterol (LDL-C), apolipoprotein A1 (APOA1) levels, but also profoundly verified the key role of the PTK2 gene in the pathogenesis of APL through cellular experiments (Graphic abstract). The results indicated that high expression of PTK2 in APL cells significantly promoted cell proliferation and migration and upregulated LDL and fibrinogen (FIB) levels while knocking out PTK2 markedly inhibited these functions, resulting in reduced cell proliferation, decreased migratory capability, and increased apoptosis rate. These findings suggest that PTK2 plays an important role in regulating lipid metabolism and promoting the proliferation and migration of APL cells and may be one of the key molecules in the pathogenesis of APL. Furthermore, the results of this study further demonstrated that lipid metabolism abnormalities in blood lipid parameters are closely related to the risk stratification, bleeding tendency, and prognosis of APL. Elevated TG levels are closely related to the pathogenesis of APL and may be involved through abnormal expression of PTK2. Lower APOA1 levels are significantly associated with a higher risk of bleeding, while lower LDL-C levels are correlated with poorer prognosis. Lactate dehydrogenase (LDH) levels are also significantly positively correlated with APL’s occurrence and risk stratification. These findings provide new perspectives and a theoretical basis for APL’s clinical diagnosis, early prevention, and treatment strategies. This study systematically demonstrated for the first time that lipid metabolism indicators can be used for risk stratification and prognosis assessment of APL, providing strong theoretical support for guiding the clinical management of APL through monitoring serum lipid levels. Meanwhile, PTK2 and other molecules related to lipid metabolism may become potential intervention targets in future APL treatments. Despite the innovative nature of this study, the sample size is relatively limited, including only cases from a single center, which may restrict the generalizability of the results. In addition, this study is observational and cannot establish causal relationships between lipid metabolism abnormalities and APL, thus requiring larger-scale multicenter prospective studies and further experimental validation to confirm these findings. Future research should focus on a more in-depth exploration of lipid metabolism regulation and the pathogenesis of APL, especially the specific mechanisms of elevated TG levels and the role of PTK2 in APL. Moreover, multicenter prospective studies and clinical trials will help verify the clinical effectiveness of blood lipid levels as diagnostic and prognostic indicators for APL. By developing treatments or drugs targeting lipid metabolism abnormalities, combined with personalized treatment plans, it is hoped that APL patients’ survival rate and quality of life can be further improved.

## Data Availability

The original contributions presented in the study are included in the article/**Supplementary Material**. Further inquiries can be directed to the corresponding author.

## Supplementary material

Supplementary Figure 1 Flow chart for inclusion and exclusion of APL patients and healthy controls.

Supplementary Figure 2 Efficiency of PTK2 gene knockdown in APL cells using siRNA. **(A)** NB4 and **(B)** HL-60 cells were subjected to knockdown of the PTK2 gene using three different siRNAs (siPTK2#1, siPTK2#2, siPTK2#3). The mRNA expression levels of the PTK2 gene were detected by qPCR. The results showed that siPTK2#3 had the highest knockdown efficiency, hence siPTK2#3 was selected for subsequent experiments involving PTK2 gene knockdown. The experimental results are presented in a bar graph, with relative expression levels normalized to an internal reference gene. Data are presented as the mean ± standard deviation of three independent experiments, and statistical analysis was performed using a t-test.
