# Supplementary figures and images for: Prognostic value of serum lipids in newly diagnosed acute promyelocytic leukemia
Source: Front Oncol. 2025 Feb 18;15:1522239. doi: 10.3389/fonc.2025.1522239 (PMC11876187; doi:10.3389/fonc.2025.1522239)

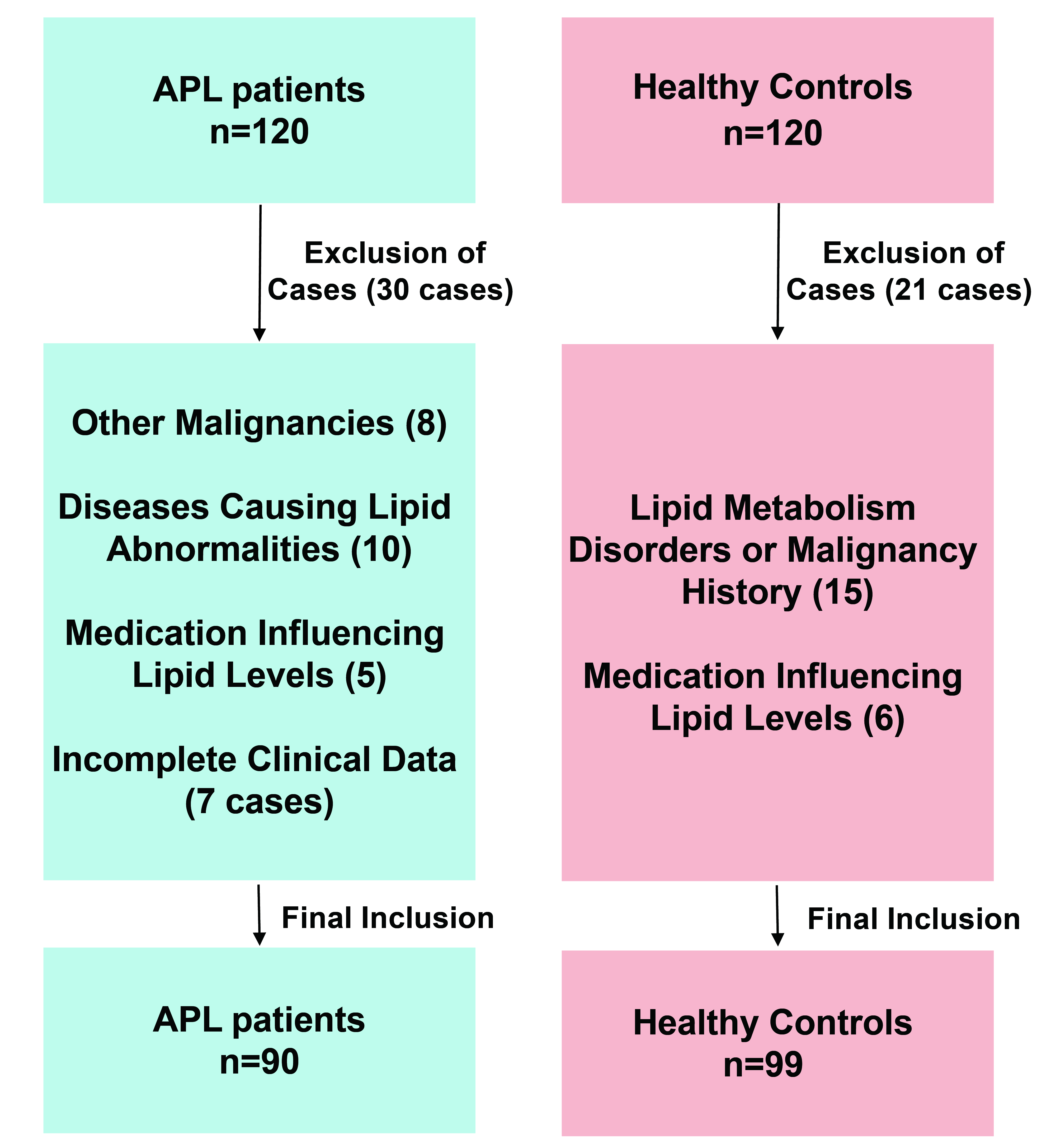

Supplement: Supplementary Figure 1 — Flow chart for inclusion and exclusion of APL patients and healthy controls. [file Image1.jpeg]

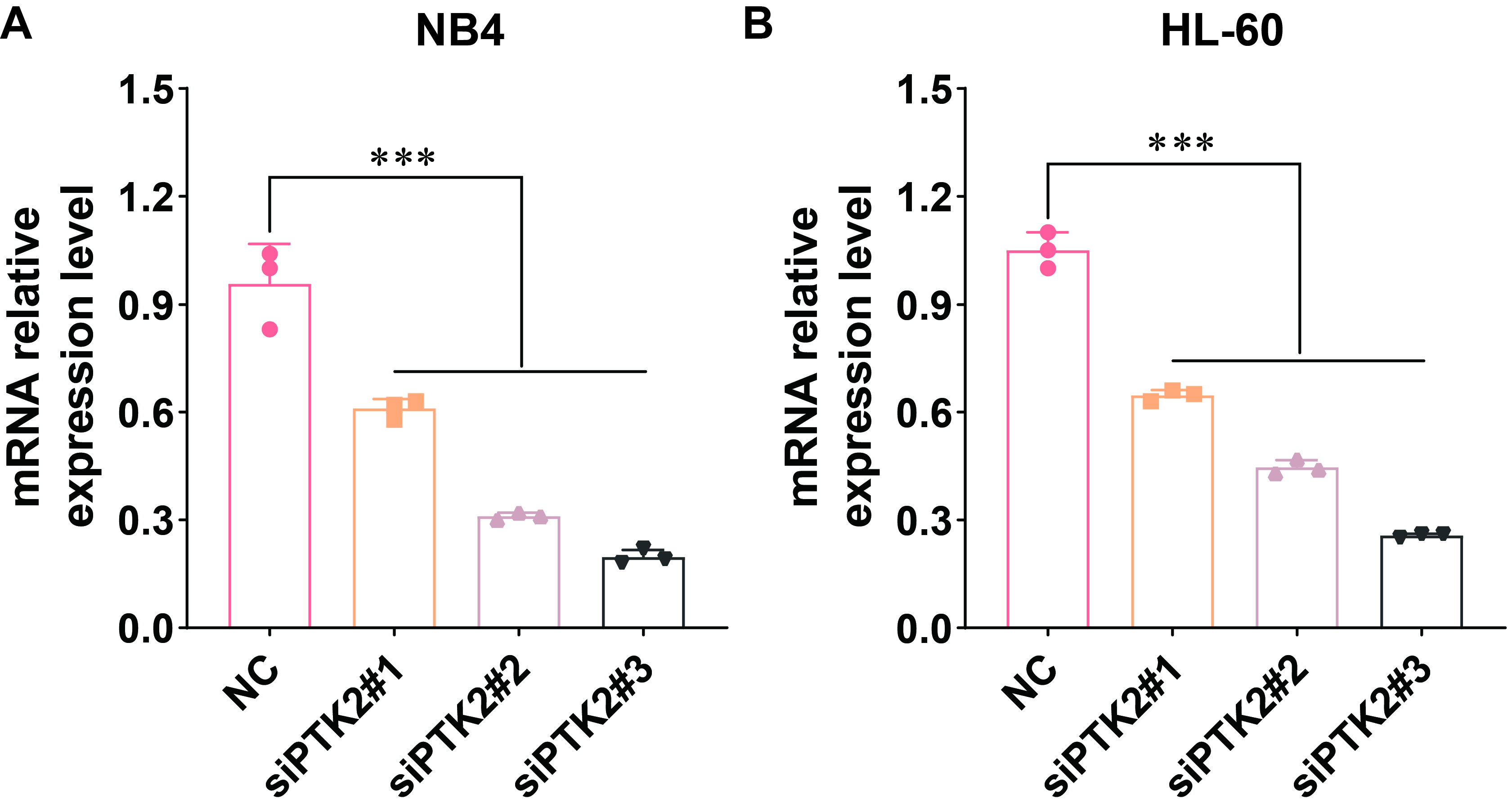

Supplement: Supplementary Figure 2 — Efficiency of PTK2 gene knockdown in APL cells using siRNA. (A) NB4 and (B) HL-60 cells were subjected to knockdown of the PTK2 gene using three different siRNAs (siPTK2#1, siPTK2#2, siPTK2#3). The mRNA expression levels of the PTK2 gene were detected by qPCR. The results showed that siPTK2#3 had the highest knockdown efficiency, hence siPTK2#3 was selected for subsequent experiments involving PTK2 gene knockdown. The experimental results are presented in a bar graph, with relative expression levels normalized to an internal reference gene. Data are presented as the mean ± standard deviation of three independent experiments, and statistical analysis was performed using a t-test. [file Image2.jpeg]
