# Supplementary material for: Prognostic value of serum lipids in newly diagnosed acute promyelocytic leukemia
Source: Front Oncol. 2025 Feb 18;15:1522239. doi: 10.3389/fonc.2025.1522239 (PMC11876187; doi:10.3389/fonc.2025.1522239)
Supplement: Supplementary file 3 [file Table1.docx]

**Supplementary Table 1.** Summary of the modified WHO bleeding scale

| **Bleeding Grade** | **Examples** |
| --- | --- |
| **Grade 0** | No bleeding |
| **Grade 1** | Oropharyngeal bleeding ≤30 min in 24 h  Epistaxis ≤30 min in previous 24 h  Petechiae of oral mucosa or skin  Purpura ≤1 inch in diameter  Spontaneous hematoma in soft tissue or muscle  Positive stool occult blood test  Microscopic hematuria or hemoglobinuria  Abnormal vaginal bleeding (spotting) |
| **Grade 2** | Epistaxis >30min in 24 h  Purpura >1 inch in diameter  Joint bleeding  Melanotic stool  Hematemesis  Gross/visible hematuria  Abnormal vaginal bleeding (more than spotting)  Hemoptysis  Visible blood in body cavity fluid  Retinal bleeding without visual impairment  Bleeding at invasive sites |
| **Grade 3** | Bleeding requiring red blood cell transfusion over routine transfusion needs  Bleeding associated with moderate hemodynamic instability |
| **Grade 4** | Bleeding associated with severe hemodynamic instability  Fatal bleeding  CNS bleeding on imaging study with or without dysfunction |

Abbreviations: WHO: World Health Organization; CNS: central nervous system
