# Supplementary material for: Prognostic value of serum lipids in newly diagnosed acute promyelocytic leukemia
Source: Front Oncol. 2025 Feb 18;15:1522239. doi: 10.3389/fonc.2025.1522239 (PMC11876187; doi:10.3389/fonc.2025.1522239)
Supplement: Supplementary file 4 [file Table2.docx]

Supplementary Table 2

Risk classification of APL

| Low-risk group | Middle-risk group | High-risk group |
| --- | --- | --- |
| WBC＜10×109/L  PLT＞40×109/L | WBC＜10×109/L  PLT≤40×109/L | WBC≥10×109/L |

Reference：

[Chinese guidelines for diagnosis and treatment of acute promyelocytic leukemia(2018)]. Zhonghua Xue Ye Xue Za Zhi, 2018. **39**(3): p. 179-183.
