# Supplementary material for: Prognostic value of serum lipids in newly diagnosed acute promyelocytic leukemia
Source: Front Oncol. 2025 Feb 18;15:1522239. doi: 10.3389/fonc.2025.1522239 (PMC11876187; doi:10.3389/fonc.2025.1522239)
Supplement: Supplementary file 5 [file Table3.docx]

Supplementary Table 3

Single factor analysis of APL and non-APL

|  | B | Wals | p-value | Crude OR (95% CI） |
| --- | --- | --- | --- | --- |
| Gender(N) | -0.82 | 7.48 | **0.006^*^** | 0.441(0.246，0.793） |
| BMI(Kg/m2) | 0.14 | 7.30 | **0.007^*^** | 1.146(1.038，1.265） |
| ALT(U/L) | 0.04 | 11.04 | **0.000^*^** | 1.035(1.014，1.056） |
| AST(U/L) | 0.06 | 12.33 | **0.000^*^** | 1.06(1.026，1.094） |
| ALP(U/L) | -0.01 | 0.97 | 0.33 | 0.995(0.985，1.005） |
| LDH(U/L) | 0.03 | 33.64 | **0.000^*^** | 1.025(1.016，1.033） |
| TC (mmol/L) | -0.01 | 0.35 | 0.55 | 0.992(0.967，1.018） |
| TG (mmol/L) | 1.00 | 24.69 | **0.000^*^** | 2.725(1.835，4.046） |
| HDL-C (mmol/L) | -1.17 | 6.45 | **0.01^*^** | 0.311(0.126，0.766） |
| LDL-C (mmol/L) | -0.53 | 4.71 | **0.03^*^** | 0.589(0.365，0.95） |
| Apo A1(g/L) | -0.71 | 2.34 | 0.13 | 0.492(0.198，1.222） |
| ApoB(g/L) | 0.95 | 2.85 | 0.09 | 2.582(0.858，7.768） |
| Cr(μmol/L) | 0.02 | 5.59 | **0.02^*^** | 1.024(1.004，1.044） |
| UA(μmol/L) | 0.00 | 5.84 | **0.02^*^** | 0.996(0.993，0.999） |

ALT: Alanine aminotransferase; AST: Aspartate aminotransferase; ALP: Alkaline phosphatase; CR: Creatinine; UA: Uric acid; LDH: Lactate dehydrogenase; TC: Total cholesterol; TG: Triglyceride; HDL-C: High density lipoprotein cholesterol; LDL-C: Low density lipoprotein cholesterol; Apo A1: Apolipoprotein A1; ApoB: Apolipoprotein B; *:P＜0.05
