# Supplementary material for: Prognostic value of serum lipids in newly diagnosed acute promyelocytic leukemia
Source: Front Oncol. 2025 Feb 18;15:1522239. doi: 10.3389/fonc.2025.1522239 (PMC11876187; doi:10.3389/fonc.2025.1522239)
Supplement: Supplementary file 6 [file Table4.docx]

Supplementary Table 4

Comparison of different risk stratification of APL

|  | High risk (18) | Medium risk (53) | Low risk (19) | P |
| --- | --- | --- | --- | --- |
| PT (s) | 13.4（12.5,16.6） | 13.4（11.8,15.0） | 11.1（10.4,12.8） | **＜0.001^ac^** |
| APTT (s) | 26.3（21.7,29.4） | 27.2（23.7,33.6） | 27.2（20.9,29.4） | 0.732 |
| FIB (g/L) | 2.37（1.50,2.76） | 1.58（0.99,2.16） | 1.06（0.72,1.55） | **0.001^c^** |
| ALT (U/L) | 25（19,56） | 20.5（14.3,38.0） | 21（12,31） | **0.021^c^** |
| AST (U/L) | 27（16,32.5） | 24（18.5,37.0） | 19（16,24） | 0.418 |
| ALP (U/L) | 80（69.5,111.5） | 81（64.5,103.3） | 79（70,87） | 0.435 |
| LDH (U/L) | 390（245,693） | 254（189.5,376.5） | 226（144,314） | **＜0.001^abc^** |
| TC (mmol/L) | 4.14（3.21,5.27） | 4.33（3.93,4.87） | 4.0（3.7,4.8） | 0.98 |
| TG (mmol/L) | 3.63（1.51,9.32） | 1.83（1.02,2.635） | 1.95（1.25,2.96） | **0.026^c^** |
| HDL-C (mmol/L) | 0.88（0.55,1.01） | 0.93（0.83,1.02） | 1.59（0.96,1.71） | **＜0.001^ac^** |
| Apo A1(g/L) | 1.15（0.93,1.48） | 1.04（0.93,1.28） | 1.12（0.94,1.69） | 0.611 |
| ApoB (g/L) | 0.77（0.67,0.94） | 0.93（0.773,0.990） | 0.90（0.79,1.04） | 0.628 |
| Cr (μmol/L) | 49（48.5,63.2） | 64.5（56.0,73.0） | 55.1（42.0,71.0） | 0.139 |
| LDL-C (mmol/L) | 2.28±0.56 | 2.31±0.59 | 2.47±0.68 | 0.53 |
| UA (μmol/L) | 230.78±45.47 | 258.79±93.6 | 244.84±91.69 | 0.47 |

The a、b、c were low risk - medium risk, medium risk - high risk and low risk - high risk groups；*:P＜0.05

PT: Prothrombin time; APTT: Partial prothrombin time; FIB: fibrinogen; ALT: Alanine aminotransferase; AST: Aspartate aminotransferase; ALP: Alkaline phosphatase; CR: Creatinine; UA: Uric acid; LDH: Lactate dehydrogenase; TC: Total cholesterol; TG: Triglyceride; HDL-C: High density lipoprotein cholesterol; LDL-C: Low density lipoprotein cholesterol; Apo A1: Apolipoprotein A1; ApoB: Apolipoprotein B;
