# Supplementary material for: Prognostic value of serum lipids in newly diagnosed acute promyelocytic leukemia
Source: Front Oncol. 2025 Feb 18;15:1522239. doi: 10.3389/fonc.2025.1522239 (PMC11876187; doi:10.3389/fonc.2025.1522239)
Supplement: Supplementary file 7 [file Table5.docx]

Supplementary Table 5

Comparison of APL in different bleeding tendencies

|  | Low bleeding risk（47） | High blood risk（43） | P |
| --- | --- | --- | --- |
| PT (s) | 12.5（11.3,15.3） | 13.4（12.15,14.4） | 0.583 |
| APTT(s) | 26.6（22.1,30.45） | 29.4（25.55,33.25） | 0.272 |
| D-dimer (mg) | 4.22（3.19，14.25） | 3.78（2.955,15.30） | 0.654 |
| WBC (10*9/L) | 1.63（0.945,4.395） | 1.34（0.865,4.525） | 0.199 |
| Hg (g/L) | 93（82.5,114.5） | 65（52.5,78.5） | **＜0.001^*^** |
| PLT (10*9/L) | 29（15,54.5） | 21（7.5,31） | **0.002^*^** |
| ALT (U/L) | 21（16,38.5） | 21（11,25.5） | 0.897 |
| AST (U/L) | 22（17,32.5） | 24（16.5,38.5） | 0.405 |
| ALP (U/L) | 82（70.5,100） | 79（61.5,100） | 0.467 |
| LDH (U/L) | 227（152.5,359） | 265（185,415） | 0.518 |
| TC (mmol/L) | 4.42（3.96，5.265） | 4.04（3.685,4.695） | 0.436 |
| TG (mmol/L) | 2.06（1.435,2.975） | 1.5（0.97,2.275） | 0.272 |
| HDL-C (mmol/L) | 0.99（0.93,1.6） | 0.87（0.79,0.96） | 0.200 |
| Apo A1 (g/L) | 1.22（0.965,1.435） | 1.01（0.845,1.11） | **0.016^*^** |
| ApoB (g/L) | 0.94（0.785,1.055） | 0.79（0.715,0.96） | 0.448 |
| Cr (μmol/L) | 61（48.5,67.5） | 59（48,76） | 0.316 |
| LDL-C (mmol/L) | 2.41±0.63 | 2.26±0.57 | 0.251 |
| UA(μmol/L) | 255.53±76 | 244.47±95 | 0.543 |

WBC: white blood cell; Hb: hemoglobin; PLT: platelets; PT: Prothrombin time; APTT: Partial prothrombin time; FIB: fibrinogen; ALT: Alanine aminotransferase; AST: Aspartate aminotransferase; ALP: Alkaline phosphatase; CR: Creatinine; UA: Uric acid; LDH: Lactate dehydrogenase; TC: Total cholesterol; TG: Triglyceride; HDL-C: High density lipoprotein cholesterol; LDL-C: Low density lipoprotein cholesterol; Apo A1: Apolipoprotein A1; ApoB: Apolipoprotein B; *:P＜0.05
